# Relative age effects on speed trials in Brazilian athletics

**DOI:** 10.1186/s13102-023-00629-z

**Published:** 2023-02-11

**Authors:** Rui Barboza-Neto, Hadi Nobari, Felipe J. Aidar, Paulo Francisco Almeida-Neto, Ana Filipa Silva, Radamés Maciel Vítor Medeiros, Filipe Manuel Clemente, Victor Sabino de Queiros, Dihogo Gama de Matos, Luiz Felipe da Silva, Georgian Badicu, Paulo Moreira Silva Dantas, Breno Guilherme de Araújo Tinôco Cabral

**Affiliations:** 1 Physical Activity and Health, Departament of Physical Education, Federal University of Rio Grande do Norte, Natal, RN Brazil; 2 Department of Motor Performance, Faculty of Physical Education and Mountain Sports, Transilvania University of Braşov, 500068 Brasov, Romania; 3 Department of Exercise Physiology, Faculty of Educational Sciences and Psychology, University of Mohaghegh Ardabili, Ardabil, 56199-11367 Iran; 4 Faculty of Sport Sciences, University of Extremadura, 10003 Cáceres, Spain; 5 Department of Physical Education, Federal University of Sergipe (UFS), São Cristovão, 49100-000 Brazil; 6 Escola Superior Desporto e Lazer, Instituto Politécnico de Viana do Castelo, Rua Escola Industrial e Comercial de Nun’ Álvares, 4900-347 Viana do Castelo, Portugal; 7 Research Center in Sports Sciences, Health Sciences and Human Development (CIDESD), Polytechnic Institute of Maia, 5001-801 Maia, Vila Real, Portugal; 8 Rio Grande do Norte University Center (UNIRN), Natal, RN Brazil; 9 Instituto de Telecomunicações, Delegação da Covilhã, 1049-001 Lisbon, Portugal; 10 Cardiorespiratory and Physiology of Exercise Research Laboratory, Faculty of Kinesiology and Recreation Management, University of Manitoba, Winnipeg, MB R3T 2N2 Canada; 11 Department of Physical Education and Special Motricity, Faculty of Physical Education and Mountain Sports, Transilvania University of Braşov, 500068 Brasov, Romania

**Keywords:** Birthday distribution, Track and field, Youth sport, Gender, Sports training, Talent selection

## Abstract

**Background:**

Relative age effect (RAE) is a concept related to the possible advantage that older athletes would have over younger ones within the same category. Although many studies have approached this subject in individual sports, there are few clippings by events within the sport. More detailed analyses are necessary for a better understanding of how RAE behaves in sports, especially in athletics, the subject of this study. The objective of this study was to analyze the RAE on speed in track and field events as a whole, separating the flat races from the hurdles races.

**Methods:**

The Brazilian Ranking of Brazilian Athletics Confederation was used for data analysis, and the sample was composed of the 50 best-placed marks in the ranking of speed events in athletics in the categories Under(U)-16 and U-18 (female and male). Statistical analysis was calculated by chi-square, and the effect size was checked by Cramer’s V. Likelihood-ratio test (L-Ratio) assessed the probability of the RAE occurring in the total sample and by age groups.

**Results:**

In the total sample the results pointed to the emergence of RAE in males in both categories (U-16: *p* < 0.001; V: 0.13; L-Ratio: 3.64, U-18: *p* < 0.001; V: 0.13; L-Ratio: 3.80), whereas in females no such effect was found in any category (U-16: *p* = 0.6; V: 0.09; L-Ratio: 0.09, U-18: *p* = 0.6; V: 0.07; L-Ratio: 0.12). When the results were separated by type of event, there was only a RAE in the shallow event in the U-18 female category (*p* = 0.3; V: 0.11; L-Ratio: 8.72).

**Conclusion:**

The results allow us to conclude that there is a RAE in the speed trials of Brazilian athletics in the U16 and U18 categories for men, while this effect appears only in the shallow trials of the U18 category for women, indicating that the RAE has incidence when there is more participation and competition in the sport.

## Introduction

In Athletics, as in many other sports (e. g. Soccer, Swimming, Tennis, Handball, Hockey, Volleyball), World Athletics divides its categories according to certain age groups (Rule 141 of the book Competition and Technical Rules of the World Athletics, 2020). In Brazil the division is arranged as follows: U-14 (11–13 years), U-16 (14 and 15 years), U-18 (16 and 17 years), U-20 (18 and 19 years), U-23 (20–22 years). As each category is divided with a 2-year interval, the difference can reach up to 24 months between athletes of the same category, depending on the year and month of birth. Given this situation, the concept of the Relative Age Effect (RAE) arose, which is based on the unequal distribution of the birth date, whereby individuals born in the first months of a category selection year would have a competitive advantage over their peers born at the end of the year [1]. Although commonly described in the literature, this phenomenon is not referenced in all sports [2], being influenced according to their main characteristics, since some demand more strength and power, others resistance, others technique, flexibility and agility.

It is a consensus that the RAE is more common in sports that require more strength and power for sport success [3]. On the other hand, in sports that require more endurance or technique, the RAE is less found because variables of body size (height and mass), strength and power do not have as much influence on the result of the sport [4]. Therefore, it is worth noting that in athletics both events require more power and strength and those that require more technique or endurance.

Many records exist in the literature on RAE in team sports [1, 5–9], and in individual sports [4, 10–14]. In articles on team sports there is a general analysis, but there are also analyses by playing time [5] and by position of the players [15], that is, a division was made within the sport itself to analyze the RAE in more detail. In studies of individual sports, on the other hand, the results still lack consistency [16]. Not as many analyses are observed that proposed more specific divisions between types of trials or characteristics of athletes that could bring different results compared to a general analysis of each modality. Although studies have observed an upward trend in the number of publications on RAE, offering insight into publication trends, patterns, and hot spots in research on RAE in sports may help identify research frontiers and overlooked concepts for future studies in the specific area [17]. In this sense, it aroused our interest to investigate whether the RAE occurs in the speed events of Brazilian Athletics, seeking to emphasize the analyses in a general perspective and also from the division of athletes in flat races and races with barriers.

Another point still unclear in the literature is the understanding of RAE in female sports [18]. In the review article by Much and Grondin [2] and the meta-analysis by Cobley et al. [19] not enough data was found to explain why there is a lower appearance of RAE among girls. Explanations point to less competition in women's sports and earlier maturation in girls, but to the best of our knowledge there is no consensus on the issue. Brazo-Sayavera et al. [16] demonstrate the little data in the literature on women's sport and thus points out the importance of a larger and broader investigation for this category. As social contexts, participation, and sport structures differ across countries, it is important to gather RAE data from diverse locations to aid in the quest to understand this phenomenon in women's sport.

Athletics is a modality that has many races with characteristics of high prevalence in physical aspects such as strength and power. The division of several races, 23 males and 22 females in the Adult category, also observed, besides the marathon for both sexes. To categorize the events, the groups can be analyzed: (1) speed (pure and prolonged, with and without barrier), (2) middle and long distance, (3) jumping (horizontal and vertical) and (4) throwing and throwing. In some studies, found on RAE in Athletics [20], it was seen that there are differences in the incidence of RAE between events when analyzing the world ranking. A study that analyzed Spanish athletes from the Throwing and throwing group of events [21] also found difference in the patterns of appearance of RAE by events, even though such effect appeared in all the events analyzed. Other studies in Athletics [22–24] on RAE have been published with data from Europe, presenting general data and more specific analyses, such as analysis of a group of races or athletes participating in training camps. However, such studies may not reflect the reality of Brazilian Athletics, given that the sport's popularity in this country does not compare with the cited studies [25]. The study by Figueiredo et al. [25] analyzing the RAE in Brazilian athletics, although with robust data and segmented by age, gender and type of event, did not compare the most practiced and the least practiced events within the sport.

Because of the above and the evidence in the literature, the work is focused on analyzing, via Brazilian Ranking, the RAE in speed races of Athletics as a whole and separating between the shallow races and races with barriers. The choice for the group of speed events was based on the fact that they require a lot of strength and power [20], abilities that suggest the onset of RAE. The division between the flat races and the hurdles races was used to identify possible changes in the RAE trend, since the flat races are more practiced and disputed than the hurdles races in Brazil. In this sense, the hypothesis of this study points to the premise that with the division of the races between flat and high hurdles, the RAE will be more evident in the flat races due to their greater number of practitioners.

## Material and methods

### Participants

The analysis was composed of 903 results of athletes (male and female) ranked in the speed events of the categories Under-16 (n = 401) and Under-18 (n = 502). The 50 best ranked results in the 2019 Brazilian Ranking of the Brazilian Athletics Confederation (CBAt) of each race were considered. The analyzed races are part of the group of speed races. In the under-16 category, the 75 m dash (men and women), 250 m dash (men and women), 80 m hurdles (women), 100 m hurdles (men), and 300 m hurdles (men and women) were included in the analysis. In the under-18 category the 100 m dash (men and women), 200 m dash (men and women), 400 m dash (men and women), 100 m hurdles (women), 110 m hurdles (men) and 400 m hurdles (men and women) were included. The 75 m dash (men’s under-16) and the 100 m and 200 m dash (women’s under-18) had an extra athlete in each due to a tie in the last ranking mark, so the 51 best-placed athletes in these races were analyzed.

### Study design

The study has a descriptive character with secondary data. The data were collected from the website of the Brazilian Athletics Confederation (https://www.cbat.org.br), publicly available. The ranking is formed by collecting the best result of each athlete during the year 2019. The competitions considered for the ranking may be regional, national, or international, and the athlete’s best result is always used. It is worth mentioning that in speed races there is the incidence of wind, the measurement of its speed is necessary to validate or not possible records. Only the marks with wind speed equal to or less than 2 m/s were considered, wind speed valid for World Athletics homologated records. In short, the sample comprised the best results of Brazilian athletes in speed races in their category. Another important observation is that within the ranking some athletes in the under-16 category can participate in the under-18 category, as well as athletes can participate in more than one race, and the repetition of names existed in the sample. The option to repeat names in the sample was because there was no way to decide which race to take the repeated name from. Moreover, the methodology for analyzing RAE is the quarter of birth, regardless of the year of birth, so if an athlete from a lower category plays in the older category, we believe his result should be considered. The choice of 2019 for the analysis was due to the Pandemic that prevented a reasonable number of competitions throughout the year of 2020, and thus may bring a limitation of the sample with a smaller number of tournaments compared to previous years.

The birth month was divided into quartiles, as proposed by Grondin [26], being First Quartile = January, February and March; Second Quartile = April, May and June, Third Quartile = July, August and September; Fourth Quartile = October, November and December. The fact of using public databases exempts this study from the analysis of a local ethics committee.

### Statistics analysis

All analyses were performed using open source software R (version 4.0.1; R Foundation for Statistical Computing^®^, Vienna, Austria) considering *p* < 0.05. The RAE contingency level was analysed using the Chi-square (χ^2^) goodness-of-fit test. Thus, we considered as “Base Column” the birth quartile data, as “Data Rows” the sex [male and female], the category [U-16 and U-18], and the results of the grouped [barrier + shallow] and segregated [individual analyses for barrier and shallow races] athletics events; we weighted the weight of the analyses with the values of the ranking position variable [from 1st to 50th place]. The effect size for the χ^2^ test analyses was verified by “Cramer’s V” considering the magnitude: Small 0.06–0.17; medium 0.18 and 0.29; and large > 0.29 [27]. We used the Likelihoot Ratio (L-Ratio) test to assess the probability of the relative age effect occurring in the total sample and the U-16 and U-18 categories stratified by sex. To interpret the L-Ratio we use magnitude [24]: Values between 0 and 1 reduce the probability [0.1–0.19: Large decrease, 0.2–0.49: Moderate decrease, 0.5–0.99: Slight decrease and 1: Neutral Probability]; Values > 1 increase the probability [1.1–1.9: Neutral Probability, 2–4.9: Slight increase, 5–9.9: moderate increase and ≥ 10: Large increase].

### Comparison

We checked the difference of the contingency of relative age between the sexes [male and female] through the “U” test of “Man-Whitney”, the effect size was checked by Spearman’s “Rho” and magnitude adopted was: Small: 0.10–0.29; Medium: 0.30–0.49; Broad: 0.50–0.79; Very broad: ≥ 0.80 [28].

#### Odds ratio

To calculate the Odds Ratio (OR) of RAE affecting ranking placement, we performed logistic regression analyses using semester of birth as a dichotomous variable (semester 1: born from January to June; semester 2: born from July to December). Next, ORs were calculated with respect to quartile 1 versus quartile 4 ((Q1:Q4) [dichotomous variables]). We considered the effect of the factor’s category (U-16 and U-18) and test type (barrier and non-barrier). Finally, we checked the OR about the Quartile of birth, considering the quartiles as dichotomous variables (e.g., born in quartile 1 & Not born in quartile 1). To this end, we also considered the factors category and test type.The OR was interpreted as follows: < 1 probability of non-occurrence, = 1 null probability, > 1 probability of occurrence [24].

## Results

Table 1 shows the number of athletes in each race, showing that the athletes had similar mean chronological age in both genders, with no significant differences in age when comparing the genders (*p* > 0.9; Rho < − 0.01).

**Table 1:** Sample characterization

Events	Participants (n)		Chronological age (months)	
	Male	Female	Male	Female
*U-16*				
75-m shallow	51	50	14.98 ± 0.50	14.62 ± 0.66
250-m flat	50	50	15.09 ± 0.50	14.48 ± 0.73
80-m with barrier	–	50	–	14.61 ± 0.72
100-m with barrier	50	–	15.00 ± 0.39	–
300-m with barrier	50	50	14.93 ± 0.52	14.48 ± 0.71
*U-18*				
100-m flat	50	51	16.76 ± 0.78	16.45 ± 0.83
200-m flat	50	51	16.82 ± 0.73	16.46 ± 0.84
400-m flat	50	50	16.77 ± 0.64	16.49 ± 0.85
100-m with barrier	–	50	–	16.23 ± 0.93
110-m with barrier	50	–	16.58 ± 0.80	–
400-m with barrier	50	50	16.54 ± 0.74	16.05 ± 0.82

(n): Absolute number. ± : Mean and standard deviation

In the male sample the effect of relative age was significant for total sample, U-16 and U18, while in the female sample there was no significant effect of relative age. However, when considering the entire sample, there was a moderate probability of relative age occurring frequently in the category in question in both males and females. In the sample stratified by categories (U-16 and U 18), the probability of relative age occurring was slightly increased in the male sample and was neutral in the female sample. The contingency of relative age in the male sample was higher than the female sample (Total Sample: *p* < 0.001; Rho: 0.97, 95% CI 0.79; 0.99. U-16: *p* = 0.02; Rho: 0.91, CI 95%: 0.70, 0.93. U-18: *p* = 0.02; Rho: 0.90, 95% CI 0.73, 0.95).

When segregating the sample in subgroups, it was found relative age effect in the total sample of both sexes for the flat races, for both sexes the probability of the relative age effect occurring was moderate. However, when considering the U-16 and U-18 categories, a relative age effect was found for the male sample in both categories mentioned in the barrier and non-barrier races, and a moderate probability of the relative age effect occurring frequently in both categories. For the female sample, the effect of relative age was indicated only in the U-18 category in the shallow trials, with moderate probability of the effect of relative age occurring frequently in that category.

The contingency of relative age in the male sample was higher than in the female example in the shallow trials (Total Sample: *p* < 0.001; Rho: 0.77, 95% CI 0.59, 0.81. U-16: *p* = 0.0002; Rho: 0.81, 95% CI 0.50, 0.92. U-18: *p* < 0.001; Rho: 0.93, 95% CI 0.73, 0.95) and with the barrier (Total Sample: *p* < 0.001; Rho: 0.87, 95% CI 0.70, 0.90. U-16: *p* < 0.001; Rho: 0.91, 95% CI 0.80, 0.93. U-18: *p* < 0.0001; Rho: 0.90, 95% CI 0.87, 0.92).

## Discussion

This study's objective was to analyse the Relative Age Effect (RAE) in the speed race group of Brazilian Athletics using the 2019 national ranking. This ranking is composed of the best competition results of each athlete. We found the RAE in the two categories analyzed, under-16 and under-18 males. In the two female categories, no RAE was observed in the sample composed of all the results of the speed trials. A more detailed analysis was necessary to understand better RAE in athletics, a sport that includes several races that may or may not present differences in the division of quartiles of athletes' births. For this, in addition to analyzing the group of speed events, we separated the results from hurdle and flat races. In analyzing the results of these subgroups of events, we found a greater tendency for the RAE to appear in the flat races, both male and female.

This greater influence of the RAE in the shallow races compared to the hurdle races is opposed to that found by Brustio et. al. [20] who analyzed the top 100 in World Athletics in the U-18, U-20 and adult categories. Brustio et al. [20] verified that the RAE was more present in hurdle races. The authors explained that such races brought anthropometric characteristics (limb size and height of athletes) that further developed would provide an advantage to athletes. Our explanation for this difference in findings is precisely the competition of the events. On a world scale, participation and competition in athletics is great, and in the barriers this competition is maintained, giving the possibility of social and maturational factors influencing athlete selection. In Brazil, with about 7 thousand members of the CBAt (data from the Extranet of the Confederation), the low participation in the hurdles races seems to be the determining factor for the non-emergence of the RAE.

Grondin et al. [26], in one of the first studies on the subject, demonstrated that the quantity of athletes practicing influences the appearance of the RAE. This statement was also made in later studies [2, 4, 29], who pointed out that the RAE is more evident in culturally popular sports with more demand for the practice. Although athletics is not a popular sport in Brazil, internally some races are more sought after than others, and this can be seen in the number of athletes per race in the championships. For example, in the Under-16 and Under-18 Brazilian Track and Field Championships there were more participants in the flat races than in the hurdles races (https://www.cbat.org.br/site/?pg=132).

Another situation to highlight is that RAE is rarely found in females. A probable explanation would be the earlier maturation of girls to boys [16]. Another hypothesis is the lower demand for sports practice by girls [30]. In our study we found something along these lines. Considering the general speed trials (Table 2), the RAE is only verified in males in both categories. However, in Table 3, where we analyze the hurdle races (less practiced) separately from the flat races (more practiced), the RAE appears in the U-18 female category in the flat races. This finding suggests that the participation and competitiveness increases the probability of SAR appearing, corroborating the study of Musch and Grondin [2].

**Table 2:** Results of the contingency analysis of the RAE in the total sample and by category of both sexes

Total Sample																		
Sex	Q1		Q2		Q3		Q4		χ^2^	*p*	V	L-Ratio	Odds ratio (CI 95%)					
	N	%	N	%	N	%	N	%					Semester	Q1:Q4	Q1	Q2	Q3	Q4
Male	152	33.7	154	34.1	85	18.8	60	13.3	60.26	< 0.001*	0.18	9.67	1.02 (1.01; 1.03)	1.03 (1.01; 1.06)	1.01 (1.00; 1.04)	1.03 (1.00; 1.05	0.99 (0.85; 1.10)	1.00 (0.91; 1.31)
Female	119	26.3	127	28.1	108	23.9	98	21.7	4.26	> 0.2	0.11	6.40	1.00 (0.99; 1.02)	1.00 (0.98; 1.02)	0.90 (0.85; 1.00)	1.00 (0.95; 1.10)	1.00 (0.99; 1.03)	1.00 (0.98; 1.02)
*U-16*																		
Male	73	36.3	64	31.8	42	20,9	22	10.9	31.29	< 0.001*	0.15	3.60	1.05 (1.00; 1.03)	1.01 (1.00; 1.05)	1.02 (1.00; 1.10)	1.01 (1.00; 1.05)	1.00 (0.95; 1.05)	0.99 (0.89; 1.02)
Female	46	23	57	28.5	45	22.5	52	26	1.88	> 0.5	0.09	0.03	1.01 (0.99; 1.03)	1.02 (0.99; 1.04)	1.00 (0.95; 1.02)	1.01 (0.97; 1.05)	0.99 (0.89; 1.00)	1.00 (0.98; 1.02)
*U-18*																		
Male	79	31. 6	90	36	43	17.2	38	15.2	32.14	< 0.001*	0.15	4.00	1.02 (1.00; 1.04)	1.02 (1.01; 2.38)	1.01 (1.00; 1.05)	1.10 (1.03; 1.15)	1.00 (0.99; 1.03)	1.00 (0.95; 1.03)
Female	73	29	70	27.8	63	25	46	18.3	6.95	> 0.05	0.07	0.08	1.00 (0.98; 1.02)	0.60 (28; 1.25)	0.99 (0.80; 1.00)	1.00 (0.97; 1.05)	0.99 (0.91; 1.04)	1.00 (0.97; 1.07)

*RAE* Relative age effect, *N* Absolute number of participants. % Percentage. χ^2^: Coefficient of contingency. *V* Effect Size by “V” Cramer, *L-Ratio* Likelihoot ratio, *U-16* Under-16 category, *U-18* Under-18 category, *Q1* First quartile, *Q2* Second quartile. *Q3* Third quartile, *Q4* Fourth quartile

*It demonstrates significance at the 0.5 level

**Table 3:** Contingency analysis of the RAE in the total sample and by category between hurdles and sprint events in both sexes

Total sample																		
Sex	Q1		Q2		Q3		Q4		χ^2^	*p*	V	L-Ratio	Odds ratio (CI95%)					
	N	%	N	%	N	%	N	%					Semester	Q1:Q4	Q1	Q2	Q3	Q4
Male	104	40.3	68	26.4	48	18.6	38	14.7	39.49	< 0.001*	0.13	9.00	1.03 (1.01; 1.05)	1.06 (1.02; 1.10)	1.05 (1.01; 1.10)	1.00 (0.99; 1.05)	1.00 (0.91; 1.05)	0.98 (0.90; 1.00)
Female	72	28.6	75	29.8	58	23.0	47	18.7	8.03	< 0.05*	0.11	6.02	1.01 (1.00; 1.02)	1.11 (1.01; 2.03)	1.02 (1.00; 1.05)	1.02 (1.00; 1.03)	1.00 (0.99; 1.01)	1.00 (0.97; 1.03)
*Total sample*																		
Male	48	24.0	86	43.0	37	18.5	29	14.5	38.2	< 0.001*	0.15	9.99	1.01 (1.00; 1.03)	1.00 (0.97; 1.04)	1.00 (0.97; 1.05)	1.05 (1.00; 1.10)	0.99 (0.89; 1.02)	1.00 (0.98; 1.07)
Female	47	23.5	52	26.0	50	25.0	51	25.5	0.28	> 0.95	0.26	9.00	0.99 (1.00; 1.04)	0.55 (0.32; 1.00)	1.00 (0.98; 1.04)	0.95 (0.90; 1.00)	1.00 (0.99; 1.05)	1.00 (0.90; 1.03)
*U-16*																		
Male	43	42.6	24	23.8	22	21.8	12	11.9	19.91	< 0.001*	0.17	9.90	1.04 (1.00; 1.07)	1.04 (0.99; 1.09)	1.01 (1.00; 1.03)	1.00 (0.99; 1.01)	1.00 (0.98; 1.02)	1.00 (0.99; 1.01)
Female	24	24.0	30	30.0	24	24.0	22	22.0	1.44	> 0.5	0.11	5.00	0.99 (0.97; 1.02)	0.99 (0.77; 1.15)	1.00 (0.99; 1.07)	1.00 (0.99; 1.03)	1.00 (0.99; 1.05)	0.99 (0.96; 1.01)
*U-16*																		
Male	30	30.0	40	40.0	20	20.0	10	10	20.0	< 0.001*	0.25	9.99	1.00 (0.96; 1.02)	1.17 (0.50; 3.07)	1.00 (0.95; 1.01)	1.00 (0.96; 1.05)	1.00 (0.99; 1.01)	0.99 (0.90; 1.01)
Female	22	22.0	27	27.0	21	21.0	30	30.0	2.16	> 0.5	0.09	5.33	1.00 (0.99; 1.05)	1.00 (0.90; 1.07)	1.00 (0.99; 1.07)	1.00 (0.99; 1.05)	1.00 (0.95; 1.02)	1.00 (0.99; 1.01)
*U-18*																		
Male	61	40.7	44	29.3	26	17.3	19	12.7	28.51	< 0.001*	0.17	9.85	1.02 (1.00; 1.05)	1.07 (1.02; 1.12)	1.01 (1.00; 1.05)	1.00 (0.99; 1.02)	1.00 (0.99; 1.02)	1.00 (0.96; 1.06)
Female	48	31.6	45	29.6	34	22.4	25	16.4	8.79	< 0.05*	0.10	8.65	1.01 (1.00; 1.02)	1.64 (1.02; 3.73)	1.03 (1.00; 1.05)	1.01 (1.00; 1.02)	1.00 (0.93; 1.03)	1.00 (0.95; 1.01)
*U-18*																		
Male	18	18.0	46	46.0	17	17.0	19	19.0	23.6	< 0.001*	0.19	9.95	1.02 (1.00; 1.05)	3.08 (1.84; 11.3)	1.00 (0.95; 1.05)	1.03 (1.00; 1.10)	0.99 (0.87; 1.01)	1.00 (0.96; 1.02)
Female	25	25.0	25	25.0	29	29.0	21	21.0	1.28	> 0.7	0.10	3.00	1.00 (0.99; 1.05)	0.74 (0.25; 2.23)	0.99 (0.87; 1.02)	0.99 (0.90; 1.01)	1.00 (0.97; 1.07)	0.99 (0.89; 1.03)

: Sprint events.  : Hurdles events. *RAE* Relative age effect, *N* Absolute number of participants. % Percentage. χ^2^ Coefficient of contingency, *V* Effect Size by “V” Cramer, *L-Ratio* Likelihoot ratio, *U-16* Under-16 category, *U-18* Under-18 category, *Q1* First quartile, *Q2* Second quartile, *Q3* Third quartile, *Q4* Fourth quartile

*It demonstrates significance at the 0.5 level

To explain the change in the pattern of SAR in women when dividing less practiced from more practiced races, the concept proposed by Hancock et al. [31], who presented a theoretical model explaining the possible social agents that would be responsible for RAE, seems acceptable. In this model it is cited how parents (via the Matthew Effect), coaches (via the Pygmalion Effect) and athletes themselves (via the Galatea Effect) act as social factors for the onset of RAE. In the case of our research, we consider the latter two as a possible explanation. Athletics coaches tend to look for the event in which the athlete would have more sporting success, even if only momentarily. It is natural, and even strategic, that coaches lead younger athletes to less competitive events, not believing much in the success of these athletes in more competitive events. The Pygmalion Effect supports this attitude. To strengthen this situation even more, the Galatea Effect presents itself when the youngest athlete sees himself with greater chances of success leaving for less competitive races. The combination of these two effects may explain the non-appearance of RAE in women's hurdle races, since these races can be a second practice option for athletes whose coaches and themselves do not see chances of success in shallow races. In this sense the article by Peña-González et al. [7] deals with a relevant variable when dealing with talent selection. It is the factor that the authors call Coach Efficacy Expectation (CEE), a factor that measures each coach's confidence in an athlete's efficiency when performing a physical/sporting task. Their findings found that CEE is higher in those born in the first trimester than those born in the fourth trimester. This reinforces our explanation regarding the coaches' decision to place athletes in more competitive events that the coaches have higher expectations of.

Despite not being verified RAE in the shallow races in the under-16 in the feminine, very practised, there is a greater incidence of those born in the first semester concerning the second semester, compared to the races with hurdles, where there is more balance between the births. In this case we should also consider the explanation of Beunen et al. [32], emphasizing that at the peak of maturation in girls there is an increase in mass. According to Garlipp et al. [33], this gain in body mass is due to the increase in height and fat mass of girls, thus allowing the inference that this would not be an advantage for shallow tests in athletics.

One hypothesis for the emergence of RAE in a higher category in women may be the aforementioned social agents. Brustio et. al. [20] had difficulty explaining RAE trends in the female adult category, finding them in some events in the same group, but not others. For example, they found RAE in the weight throw and discus throw, but nothing was seen in the javelin and hammer throw. As with these authors, we also have difficulty in presenting the probable reasons for finding RAE in the under-18 category and not in the under-16. As in the shallow events of the under-16 category there is a tendency, although not statistically significant, of more children born at the beginning of the year, and in the shallow events of the under-18 category there is evidence of RAE, a greater attention and motivation over time during training due to higher expectations of coaches may be one of the explanations, since such attention would reinforce the tendency found in the under-16 becoming evidence when moving up in category, but further investigations are needed in this sense.

Although this study did not analyze the corrective adjustments proposed by Romann and Cobley [34] for sprinters, we highlight this procedure that seems to be a tool to assist coaches in analyzing athletes and considerably reduce the RAE as also presented by Brustio and Boccia [35].

The main limitation of the present study was that we did not have the possibility of analyzing the rankings of U-14 category as the Brazilian Athletics Confederation does not perform classifications for this age group. The CBAt only ranks the top 50 athletes from each event, limiting a closer analysis of other studies that analyzed the top 100 competitors from each event. In addition, this study did not seek to analyze other aspects that could affect athletic performance, such as anthropometric variables.

## Conclusions

The results presented allow us to conclude that there is a RAE in velocity events in Brazilian Athletics for males. However, considering only the shallow races, the most practiced and competed for races, the RAE, besides appearing in males in both categories analyzed, also appears in females in the under-18 category, not appearing only in the under-16 female category. Such results bring a reflection for the professionals of the Sport and federative entities about the forms of division of the competitions and types of categories of the same ones. The results obtained can also be important information to be observed by the professionals of the area in the selection, orientation and promotion of young athlete of athletics, once the most practiced races in this modality can be influenced by the possible advantage of the age when the athlete is born in the beginning of the year of selection. Thus, adopting parallel rankings considering the athletes' quarter of birth seems to be a good tool for balancing the advantages older athletes have over younger ones.

## Data Availability

The datasets generated and/or analysed during the current study are available in the CBAT repository, https://www.cbat.org.br/novo/?pagina=ranking_quadro. The datasets used and/or analysed during the current study available from the corresponding author on reasonable request.

## Abbreviations

**CBAt**: Brazilian Athletics Confederation
**CEE**: Coach efficacy expectation
**RAE**: Relative age effect
